# Identification of a novel circRNA–miRNA–mRNA regulatory axis in hepatocellular carcinoma based on bioinformatics analysis

**DOI:** 10.1038/s41598-023-30567-2

**Published:** 2023-03-06

**Authors:** Guoqiang Zhong, Yan Lin, Zansong Huang

**Affiliations:** 1 Department of Gastroenterology, The Affiliated Hospital of Youjiang Medical University for Nationalities, Baise, 533000 Guangxi China; 2 The Graduate School, Youjiang Medical University for Nationalities, Baise, 533000 Guangxi China

**Keywords:** Cancer, Computational biology and bioinformatics, Genetics, Diseases, Gastroenterology, Medical research, Oncology

## Abstract

In recent years, circular RNAs (circRNAs) have been found to play an essential regulatory role in hepatocellular carcinoma (HCC) through various mechanisms, particularly the endogenous competitive RNA (ceRNA) mechanism. Therefore, it is significant to explore the circRNAs in hepatoma. In this study, we constructed the ceRNA and survival network using Cytoscape. We also used R, Perl software, and multiple online databases and platforms, including Gene Ontology (GO) and Kyoto Encyclopedia of Genes and Genomes (KEGG), to perform overall survival, immune cell infiltration, immune checkpoints, pathway activity, and anticancer drug sensitivity analysis of the genes. Finally, the receiver operator characteristic curve (ROC) analysis was performed to identify the diagnosis value of the genes. KEGG analysis revealed the T cell receptor signaling pathway as the main enrichment pathway. A total of 29 genes related to survival and prognosis were screened out. The findings suggest that ZNF544, WDR76, ACTG1, RASSF3, E2F3, ASRGL1, and POGK are associated with multilevel immune cell infiltration. Additionally, immune checkpoint analysis screened out the ACTG1, E2F3, RASSF3, and WDR76. It was also revealed that the WDR76, E2F3, ASRGL1, and POGK mainly activated the cell cycle and DNA damage response (DDR) pathway. The results suggest that the sensitivity toward trametinib, refametinib (RDEA119), and selumetinib correlates to the expression of WDR76. ROC analysis showed that the area under the curve (AUC) of all genes in the regulatory axis was greater than 0.7. The identified hsa_circ_0000417/hsa_circ_0002688/hsa_circ_0001387--hsa-miR-199a-5p--WDR76 regulatory axis may provide new insights into the progression, clinical diagnosis, and treatment of HCC.

## Introduction

HCC is the third leading cause of cancer-related death worldwide, with high morbidity and mortality rates, particularly in Asia^1–3^. It is insidious and prone to metastasis and often detected at advanced stages due to the lack of early symptoms. Although the current treatment of HCC, such as surgical resection, liver transplantation, targeted therapy, immunotherapy, and local treatment technology, has relatively mature, these treatments are often ineffective for patients with advanced HCC. The treatments are also unable to prolong the survival of these patients^4^. Therefore, it is imperative to identify new and more effective therapeutic targets. The advent of the era of non-coding RNAs (ncRNAs) has written a new chapter in this field, and circRNAs are among the emerging members of ncRNAs^5, 6^.

The circRNAs were first discovered in a plant virus^7^. However, due to the limitation of detection technology at the time, circRNAs were primarily regarded as a by-product of conventional linear splicing, leaving their potential unexplored. Thanks to sequencing technology, their potential has been uncovered. The circRNAs are widespread in eukaryotic cells and have a closed loop structure without 5′ and 3′ ends, which confers the resistance toward RNA enzymes and stability compared with linear RNAs^8^. The circRNAs are produced by back-splicing precursor mRNAs and consist of exons, introns, or both^9, 10^. There are several forms of circRNAs exon-circRNAs (ecRNAs), intron-circRNAs (ciRNAs), and exon–intron circRNAs (eiciRNAs). CiRNAs and eiciRNAs mainly regulate the transcription and splicing in the nucleus. Additionally, ecRNAs are transported out to perform specific biological functions, including acting as miRNA and protein sponges or translating nucleotide sequences into peptides and proteins^11–13^. The circRNAs have also been associated with various diseases, including cardiovascular diseases, immune system illnesses, and tumors^14–16^.

**Figure 1:**
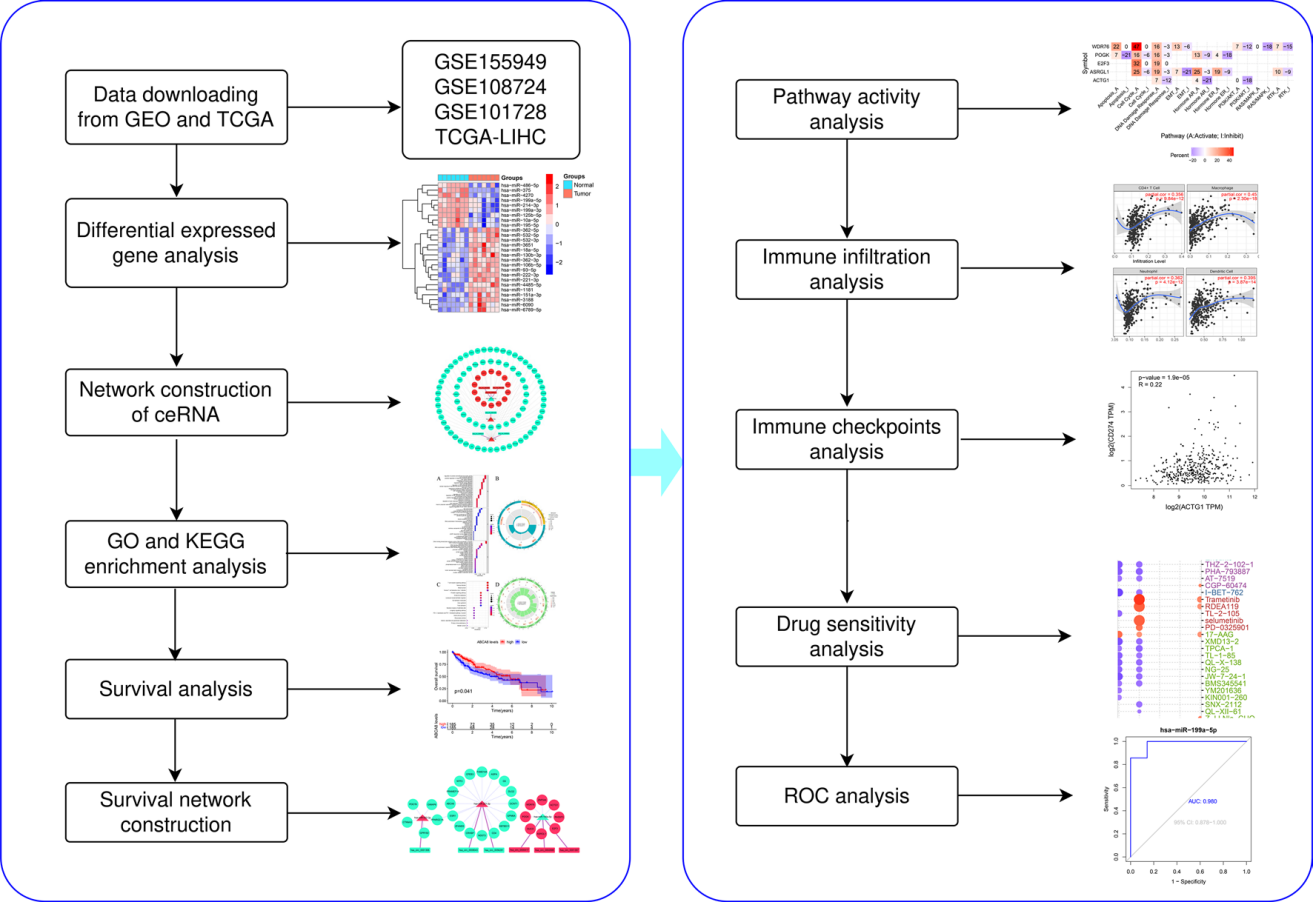
Flowchart of this study.

Evidence has emerged showing the involvement of circRNAs and ceRNA mechanism in biological processes, including liver cancer initiation, stemness maintenance of the liver cancer stem cells, and liver cancer progression. And findings suggest using the circRNA–miRNA–mRNA regulatory network as biomarkers and therapeutic targets for treating hepatocellular carcinoma^17, 18^. In recent years, bioinformatics analysis has become more and more popular in the field of scientific research^19^. Therefore, the integrated bioinformatics analysis was used in this work. We constructed a ceRNA network by analyzing differentially expressed genes according to the ceRNA mechanism. We also built the survival network and analyzed the immune cell infiltration levels, immune checkpoints, and pathway activity of the upregulated mRNAs in the survival network. The findings from this study will provide a new perspective on the pathogenesis of HCC and improve its diagnosis and treatment. Figure 1 presents the flow chart of this research.

## Results

### The downloaded data and differential gene expression analysis

**Figure 2:**
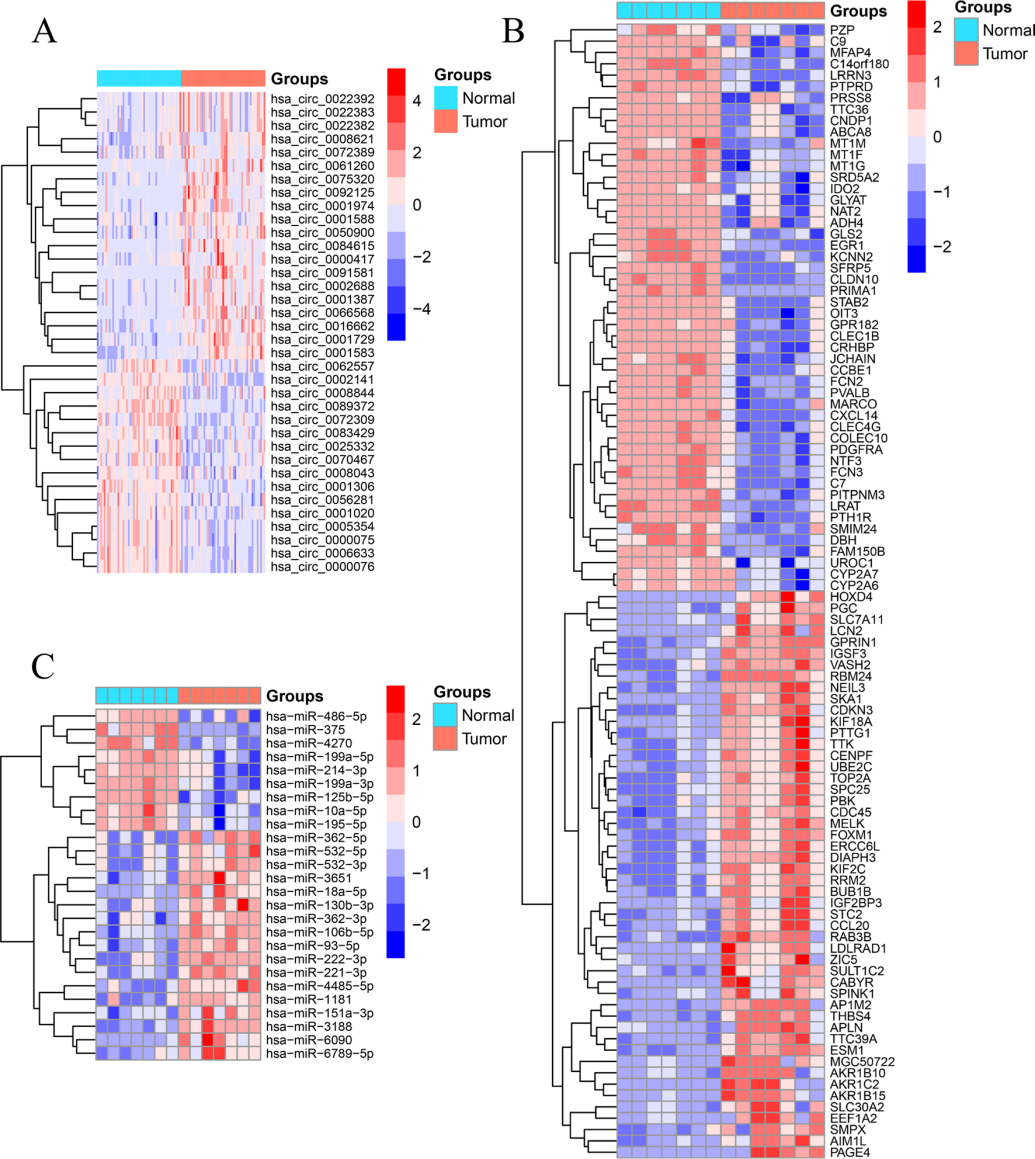
Identification of differentially expressed genes. (**A**) Heat map of the DEcircRNAs, (**B**) heat map of the DEmiRNAs, (**C**) heat map of the DEmRNAs.

We downloaded three microarray data from Gene Expression Omnibus (GEO) database (GSE155949, GSE108724, and GSE101728) and one RNA-seq data of HCC from The Cancer Genome Atlas Program (TCGA) database according to the established screening conditions. The details of the datasets are shown in Table 1. The differential gene expression analysis revealed the differential expression of 36 circRNAs (DEcircRNAs) (Fig. 2A), 26 miRNAs (DEmiRNAs) (Fig. 2B), and 2215 mRNAs (DEmRNAs) (Fig. 2C). The 36 circRNAs were used for constructing a ceRNA network.

**Table 1:** The information of the datasets in the GEO and TCGA databases.

Data types	Series	Platforms	Sample size of tumor	Sample size of normal
circRNA	GSE155949	GPL21825	49	49
miRNA	GSE108724	GPL20712	7	7
mRNA	GSE101728	GPL21047	7	7
mRNA	none	TCGA	374	50

The uniform resource locators of the three microarray datasets are https://www.ncbi.nlm.nih.gov/geo/query/acc.cgi?acc=GSE155949, https://www.ncbi.nlm.nih.gov/geo/query/acc.cgi?acc=GSE108724, https://www.ncbi.nlm.nih.gov/geo/query/acc.cgi?acc=GSE101728 respectively.

### The target prediction of circRNAs and miRNAs

**Figure 3:**
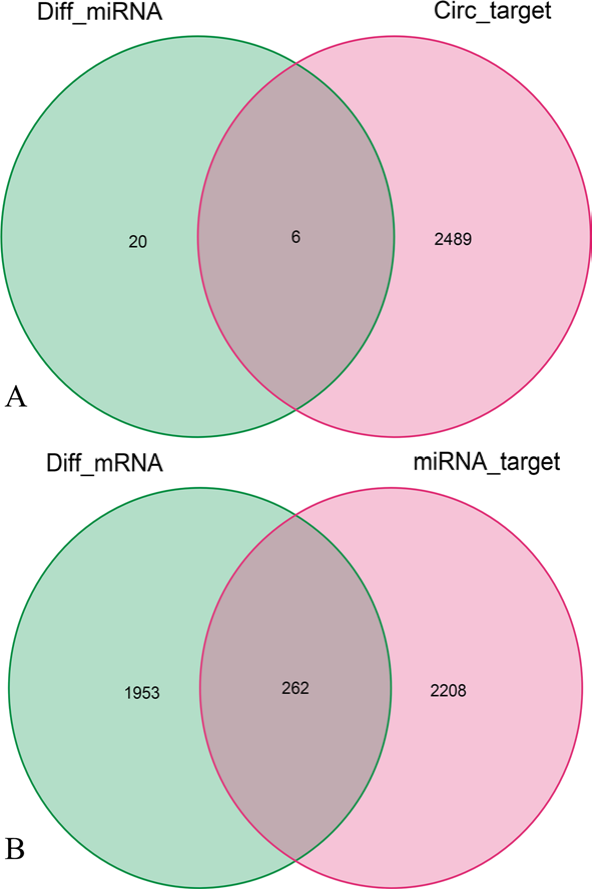
The overlapped genes of miRNA (**A**) and mRNA (**B**).

The online databases predicted a total of 2495 target genes of circRNAs, and six candidate miRNAs (Fig. 3A) after the intersection with DEmiRNAs, namely hsa-miR-375, hsa-miR-3188, hsa-miR-4270, hsa-miR-221-3p, hsa-miR-532-3p, and hsa-miR-199a-5p. Additionally, the databases predicted 2470 target genes from the six candidate miRNAs, resulting in the identification of 262 candidate mRNAs after overlapping with DEmRNAs (Fig. 3B).

### The construction of a ceRNA network

**Figure 4:**
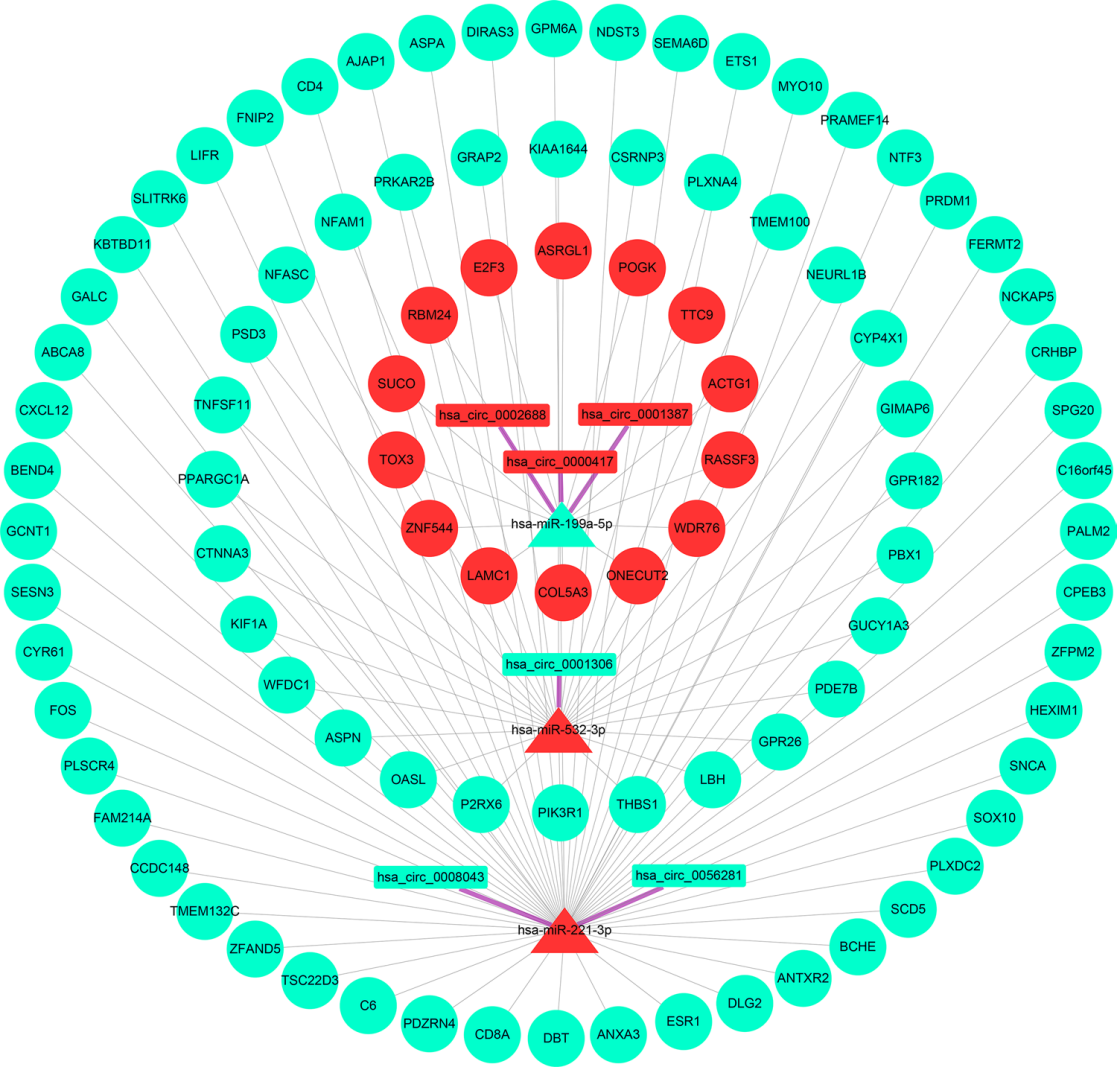
The ceRNA network.

Following the ceRNA mechanism, Perl screened the candidate genes to obtain the input files needed to construct a ceRNA network, yielding 6 circRNAs, 3 miRNAs, and 94 mRNAs. The input files were imported into Cytoscape software to build a ceRNA network containing 103 nodes and 104 edges (Fig. 4). The color of the nodes in the grid represents gene expression: red and blue represent high and low expression, respectively. Different node shapes indicate different gene types: the rectangle represents circRNAs, the triangle represents miRNAs, and the circle represents mRNAs. The lines with different colors indicate the connection between nodes: thick purple lines represent the relationship between circRNAs and miRNAs, and thin gray lines represent the relationship between miRNAs versus mRNAs.

### GO and KEGG enrichment analysis

**Figure 5:**
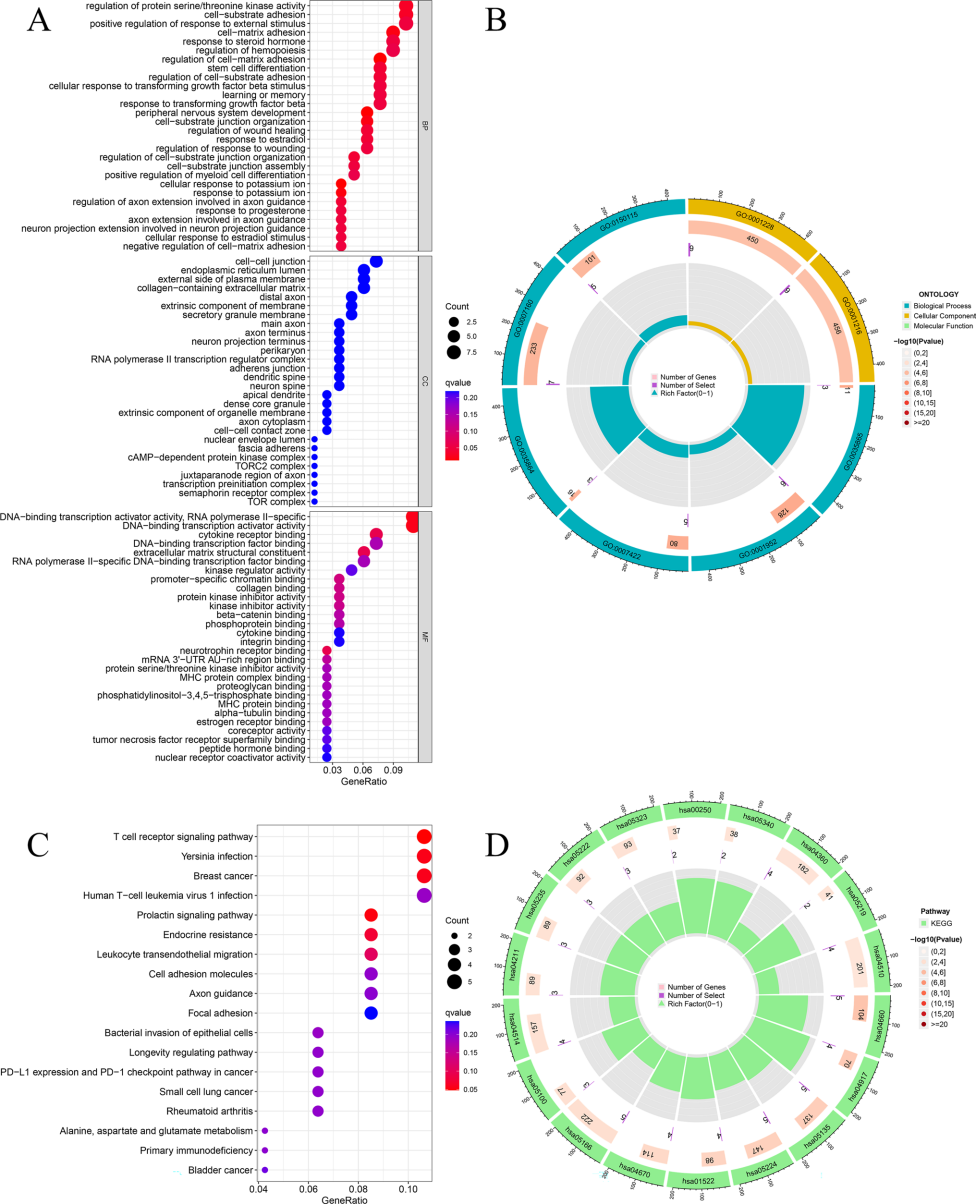
Functional enrichment analysis of the mRNAs in the ceRNA network. (**A,B**) GO enrichment analysis. (**C,D**) KEGG enrichment analysis.

The GO enrichment analysis of the mRNAs in the ceRNA network resulted in 28 GO entries. The analysis revealed the primarily enriched biological processes that include regulatory protein serine/threonine kinase activity, cell–matrix adhesion, etc. (Fig. 5A,B). KEGG enrichment analysis indicated that the T cell receptor signaling pathway was the main enriched pathway (Fig. 5C,D).

### Survival analysis and the survival network construction

**Figure 6:**
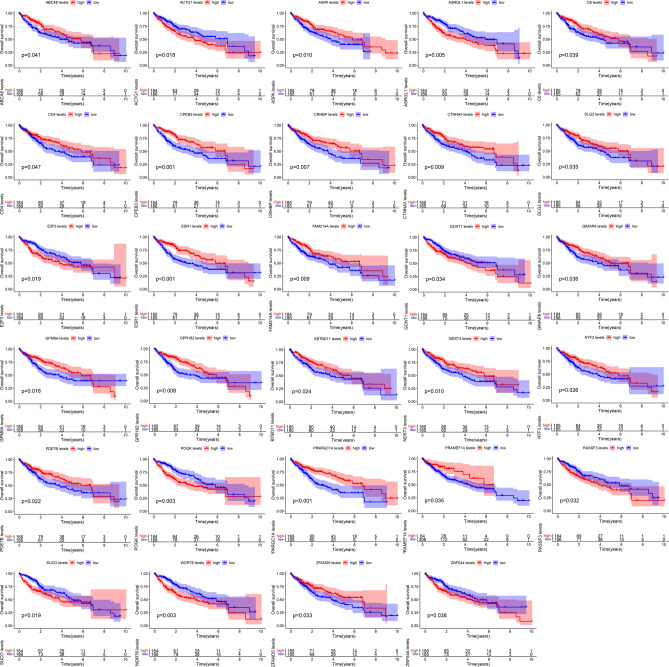
Overall survival analysis of the mRNAs in the ceRNA network.

**Figure 7:**
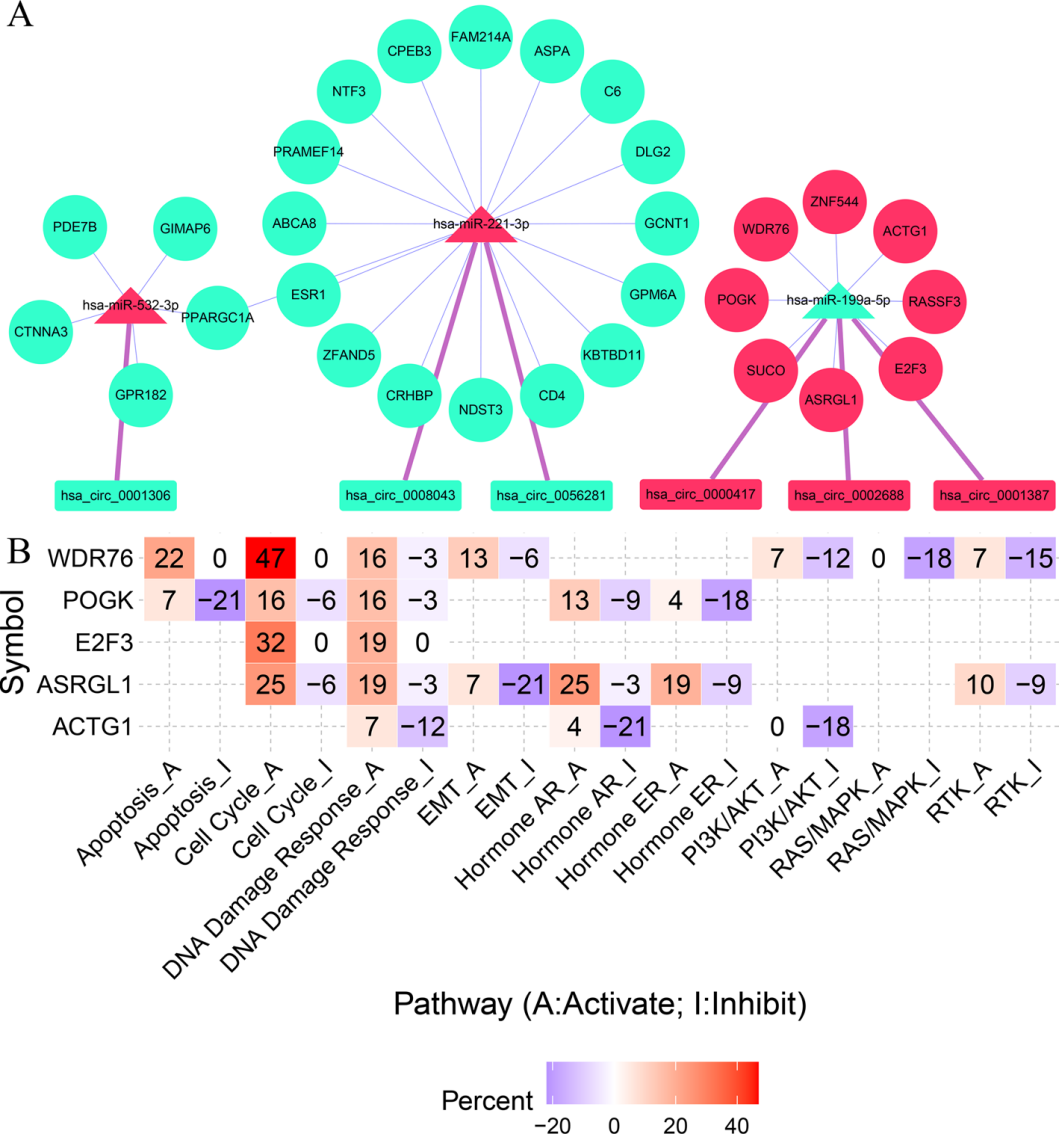
Survival network construction and pathway activity analysis. (**A**) The construction of a survival network. (**B**) Pathway activity analysis for the up-regulated mRNAs in the survival network.

Survival analysis of the mRNAs in the ceRNA network revealed 29 survival-related genes (Fig. 6). Perl was used to match the upstream genes of these 29 survival-related genes and determine their interrelationships to obtain the input files needed to construct the survival network. A survival network containing 61 regulatory axes was created using Cytoscape (Fig. 7A). The highly expressed genes of circRNAs in the survival network are presented in Table 2. The eight highly expressed mRNAs, namely ZNF544, WDR76, ACTG1, RASSF3, E2F3, ASRGL1, SUCO, and POGK from the survival network, were screened for subsequent analysis.

**Table 2:** Related information of highly expressed circRNAs in the survival network.

CircID	Location	Spliced seq length (bp)	Gene symbol	Type
hsa_circ_0000417	chr12:69644908|69656342	1599	CPSF6	ecRNA
hsa_circ_0002688	chr4:1902352|1932497	1584	WHSC1	ecRNA
hsa_circ_0001387	chr4:1902352|1936989	1703	WHSC1	ecRNA

*EcRNA* exon circRNA.

### Pathway activity analysis

Pathway activity analysis suggested that WDR76, E2F3, ASRGL1, and POGK activated the cell cycle and DDR pathway (Fig. 7B). WDR76 was identified with the most significant activation effect on the cell cycle pathway. The gene is also involved in the activation of apoptosis and epithelial-mesenchymal transition (EMT) pathway and inhibits the RAS/MAPK pathway.

### Immune cell infiltration levels and immune checkpoints analysis

**Figure 8:**
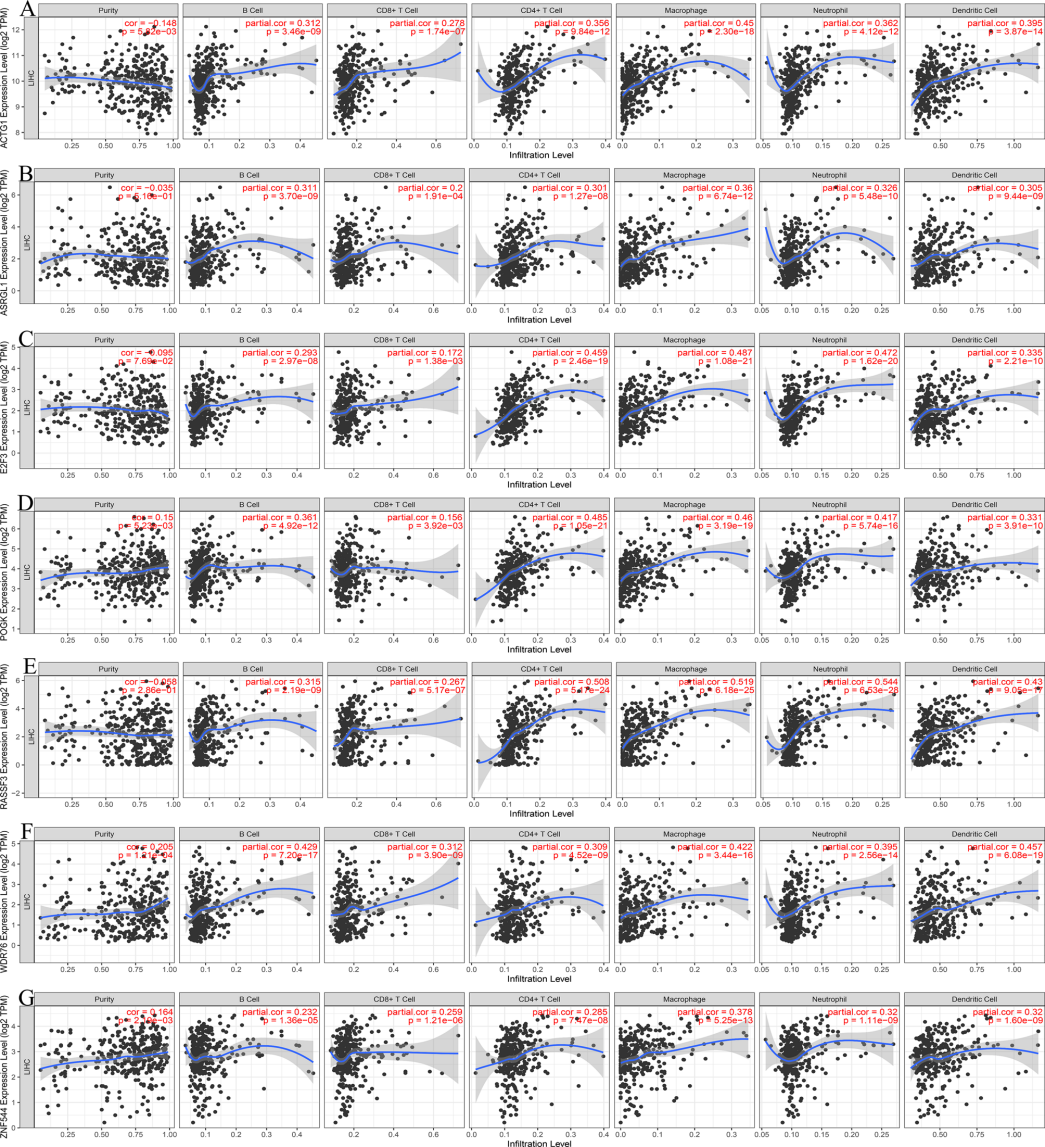
The immune cell infiltration levels analysis of the up-regulated mRNAs in the survival network. (**A**)ACTG1, (**B**) ASRGL1, (**C**) E2F3, (**D**) POGK, (**E**) RASSF3, (**F**) WDR76, (**G**) ZNF544.

**Figure 9:**
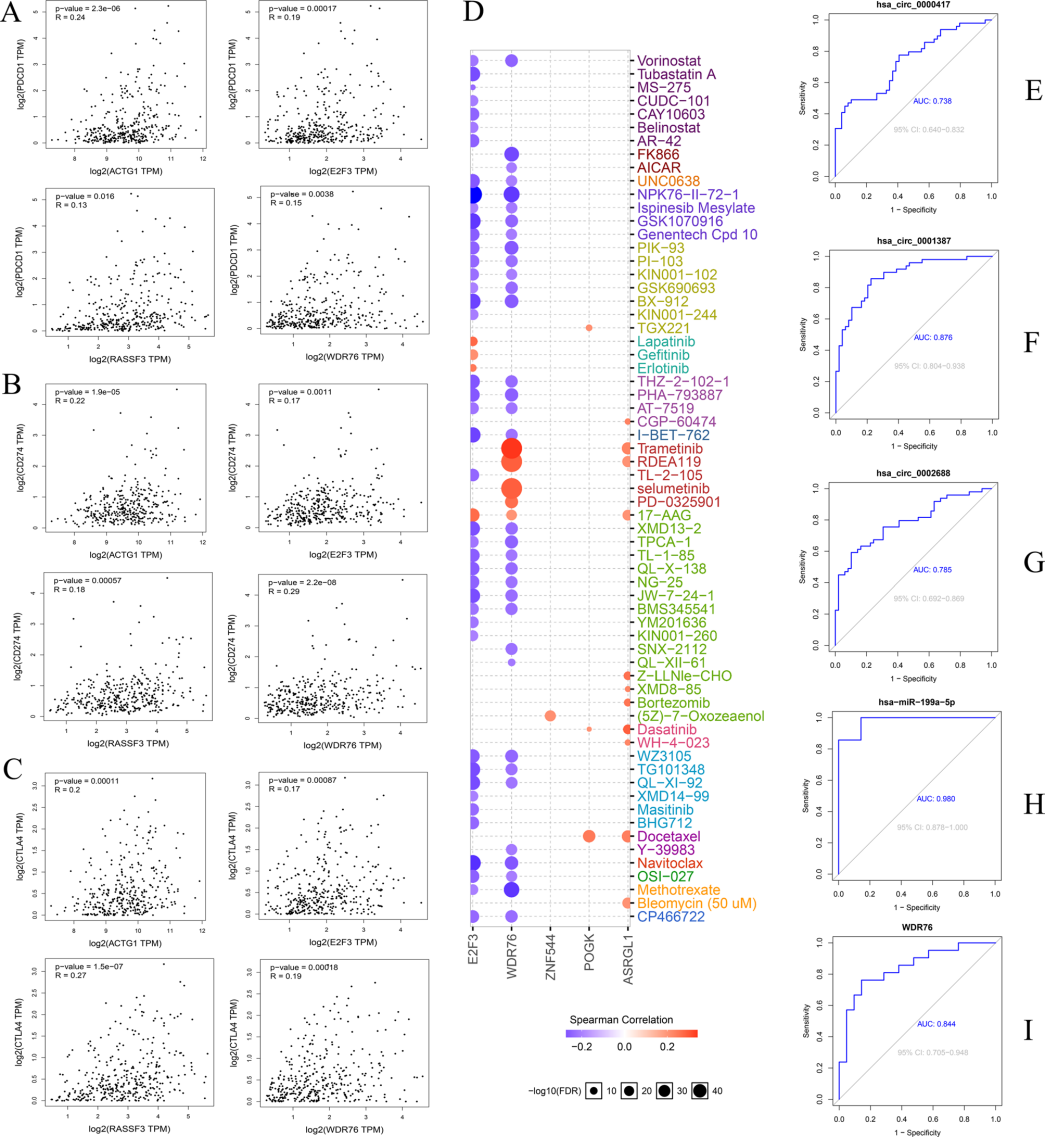
Immune checkpoints, drug sensitivity, and ROC analysis. Immune checkpoints analysis between ACTG1, E2F3, RASSF3, and WDR76 with PDCD1 (**A**), CD274 (**B**), and CTLA4 (**C**), respectively. (**D**) Drug sensitivity analysis. ROC analysis of the hsa_circ_0000417 (**E**), hsa_circ_0001387 (**F**), hsa_circ_0002688 (**G**), has-miR-199a-5p (**H**), and WDR76 (**I**).

Eight mRNAs highly expressed in the survival prognosis network were selected for immune cell infiltration analysis. The results demonstrate the significant correlation of ACTG1 (Fig. 8A), ASRGL1 (Fig. 8B), E2F3 (Fig. 8C), POGK (Fig. 8D), RASSF3 (Fig. 8E), WDR76 (Fig. 8F), and ZNF544 (Fig. 8G) with the infiltration level of B cells, CD4+ T cells, CD8+ T cells, neutrophils, macrophages and dendritic cells in the liver hepatocellular carcinoma (LIHC) microenvironment. However, the SUCO cannot be retrieved from the database. Additionally, immune checkpoint analysis revealed the positive correlation of ACTG1, E2F3, RASSF3, and WDR76 with programmed cell death 1 (PDCD1, Fig. 9A), cluster of differentiation 274 (CD274, Fig. 9B), and cytotoxic T lymphocyte-associated antigen 4 (CTLA4, Fig. 9C) in LIHC.

### Drug sensitivity and ROC analysis

**Figure 10:**
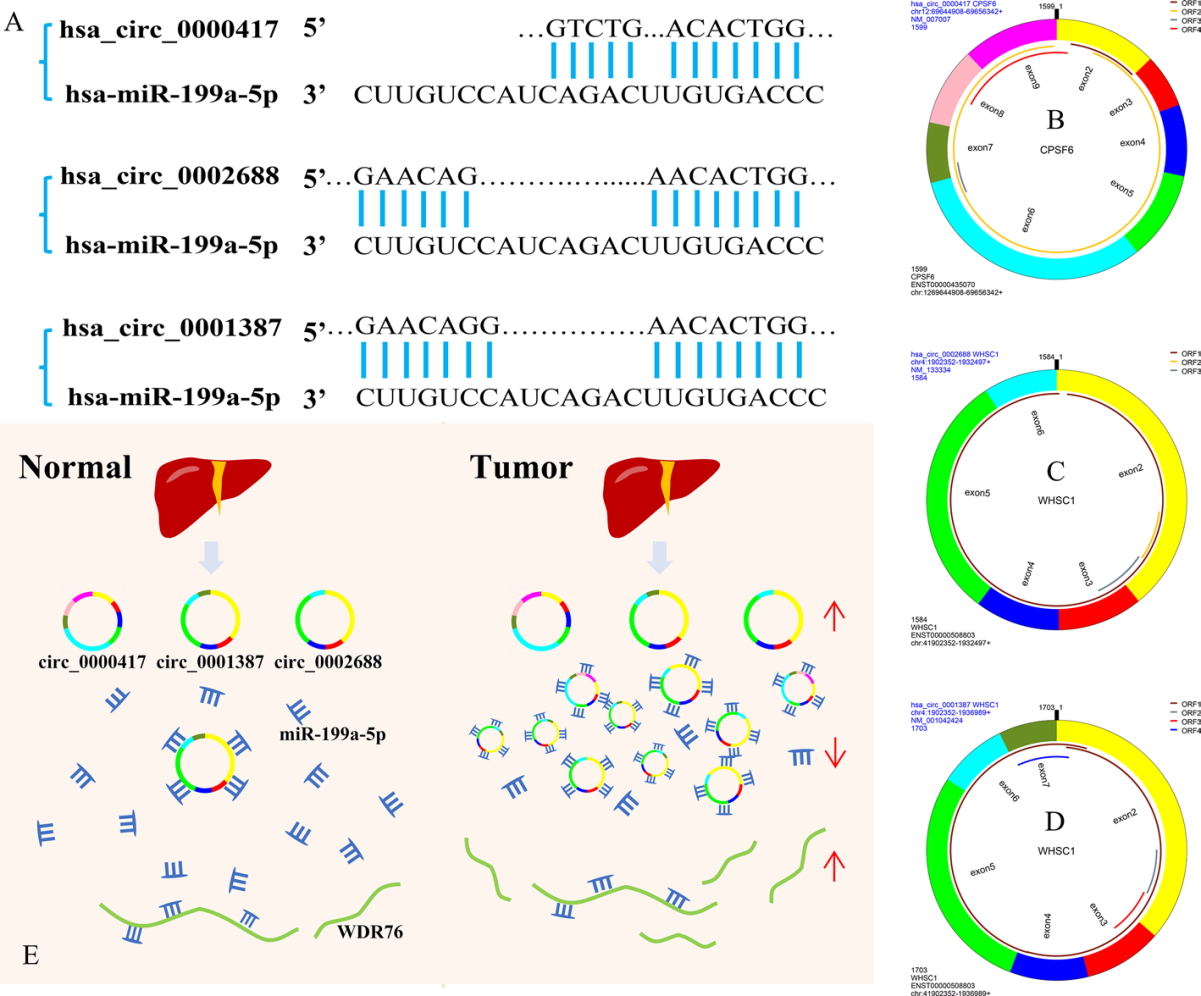
The model of the regulatory axis and the binding sites of circRNA-miRNA. (**A**) The binding sites between the hsa_circ_0000417/0001387/0002688 with has-miR-199a-5p. The structure of the hsa_circ_0000417(B)/0002688 (**C**)/0001387 (**D**), *ORF* open reading frame. (**E**) The model of the regulatory axis. The upward red arrows indicate that the upregulated expression of the corresponding RNAs, while a downward arrow indicates the opposite.

Drug sensitivity analysis showed that WDR76 was positively correlated with the sensitivity to trametinib, RDEA119, and selumetinib (Fig. 9D). The results suggest the potential role of WDR76 as a hub gene among the highly expressed genes in the survival network. Thus, we identified the hsa_circ_0000417/hsa_circ_0002688/hsa_circ_0001387-hsa_miR-199a-5p-WDR76 regulatory axis from the ceRNA network. The genes in the identified regulatory axis were subjected to ROC analysis to determine their value for HCC diagnosis. The AUCs were 0.738 (hsa_circ_0000417, Fig. 9E), 0.876 (hsa_circ_0001387, Fig. 9F), 0.785 (hsa_circ_0002688, Fig. 9G), 0.980 (hsa_miR-199a-5p, Fig. 9H) and 0.844 (WDR76, Fig. 9I), respectively. The results indicate the potential use of these genes as diagnostic markers for HCC. And, we got the potential binding sites between the circRNAs with miRNA (Fig. 10A), the structure of hsa_circ_0000417 (Fig. 10B), hsa_circ_0002688 (Fig. 10C), and hsa_circ_0001387 (Fig. 10D). Figure 10E illustrated the model of the regulatory axis. Therefore, this study revealed the connection between the regulatory axis and the survival, immune cell infiltration level, immune checkpoints, pathway activity, drug sensitivity, and HCC diagnosis of LIHC patients. Other factors that may be involved in regulating the occurrence and progression of hepatocellular carcinoma and having reference value for the diagnosis and treatment of HCC were also determined.

## Discussion

Research on various mechanisms of circRNAs in tumor cells, particularly the ceRNA mechanism, has been expanding in recent years. The ceRNA mechanism stemmed from a hypothesis that ceRNAs could compete to bind to the targeted miRNAs, decreasing the number of miRNAs and increasing the downstream mRNA targets^20^, which regulates disease. There is significant evidence supporting the role of the ceRNA mechanism in the initiation and progression of various tumors, including HCC^21–23^. We constructed a ceRNA network in this study based on the ceRNA mechanism and bioinformatics analysis. Multiple gene function analysis and identification databases ultimately determined the pivotal regulatory axis in HCC. Our findings will provide a valuable reference for the pathogenesis, diagnosis, and treatment of HCC.

Various biological processes and signaling pathways regulating tumor development have been identified. Cell matrix adhesion plays a vital role in regulating the invasiveness of different tumor cells, including invasive breast cancer^24, 25^. Additionally, the T cell receptor signaling pathway is an intracellular pathway through which T cells are partially activated to mediate an immune response. Studies have found that the cluster of differentiation 146 (CD146) combined with lymphocyte cell kinase (LCK) can promote the initiation of T cell receptor signaling and anti-tumor immune response in mice^26^. The cell cycle pathway is abnormal in almost all tumors, which may be one of the crucial reasons for the unlimited replication of tumor cells. Studies have also demonstrated that genetic variations in the cell cycle pathway may affect the survival of patients with III and IV-stage non-small-cell lung cancer (NSCLC)^27^. Cell cycle pathways may also regulate prostate cancer initiation^28^, suggesting their potential use as targets for breast cancer treatment^29^. DNA damage response is a process of cell repair following DNA damage caused by various reasons. A compromised repair process may lead to the instability of the mutant transmission genome and benefit the progress of tumor cells. A study found that apurinic apyrimidinic endodeoxyribonuclease 2 (APE2) can regulate the DDR pathway to maintain the integrity of the pancreatic cancer genome^30^. In gastric cancer, DDR can predict tumor progression and clinical prognosis^31^. These findings indicate that the ceRNA network identified in our study may promote the initiation of HCC and the invasion and progression of HCC cells, thereby deteriorating the clinical prognosis of HCC patients. The results from survival analysis also support this clinical prognostic value.

MiRNAs are short ncRNAs that can act as epigenetic regulators to promote or inhibit tumorigenesis and progression in malignant tumors such as HCC. In this study, we identified hsa-miR-199a-5p through bioinformatics analysis. Studies have found that hsa-miR-199a-5p targeting kelch-like family member 3 (KLHL3) may be involved in the occurrence and development of NSCLC^32^. The high expression of long ncRNA (lncRNA) AB209371 was found to be beneficial to the EMT of liver cancer cells and may also be the direct cause of the inactivation of hsa-miR-199a-5p. Silencing AB209371 combined with overexpression of hsa-miR-199a-5p can inhibit liver cancer metastasis and EMT of liver cancer cells, suggesting the potential use of hsa-miR-199a-5p as a target for inhibition of HCC metastasis^33^. The finding is consistent with the results obtained from our previous research. Immune cell infiltration is an integral part of the tumor microenvironment. Studies have shown that tumor-infiltrating immune cells such as B cells, CD4+ T cells, CD8+ T cells, neutrophils, macrophages, and dendritic cells can affect the efficacy of immunotherapy and the prognosis of cancer patients^34, 35^. Gao et al. found an association between the SORT1 gene and immune cell infiltration, suggesting its potential as a new biomarker for HCC prediction. Interfering with SORT1 can significantly inhibit the growth of HCC cells, indicating a potential new target for developing anticancer strategies, specifically HCC^36^. In general, immune checkpoints act as regulators of the immune system to maintain and regulate autoimmune and peripheral tissue immune processes. However, tumor cells take advantage of this mechanism to evade immunity^37^. Studies have found that multifunctional aptamer (P1/C4-bi-apt) can block CTLA4/B7 and PDCD1/CD274 signaling pathways, enhancing the immune response against HCC. The potential use of this aptamer in HCC immunotherapy was previously explored^38^. It was also found that the upregulation of immune checkpoint CD274 could increase the expression of cell division cycle-associated protein 2 (CDCA2) and predict the poor prognosis of HCC^39^. We found that ACTG1, E2F3, RASSF3, and WDR76 in the ceRNA network are closely related to the immune checkpoints, suggesting that the ceRNA network regulates the immune escape of HCC cells. Meanwhile, drug sensitivity analysis revealed an association between WDR76 and the sensitivity to trametinib, RDEA119, selumetinib, and other anticancer drugs. Prior studies have demonstrated that refametinib combined with sorafenib is more beneficial in RAS-mutant HCC patients who have lost the chance of surgery^40^. However, the therapeutic value of selumetinib combined with sorafenib for HCC is not significantly different from other monotherapy^41^. Trametinib has been found to produce a significant effect in treating ovarian cancer and high and low-grade gliomas^42, 43^. However, such an effect on HCC has yet to be reported. ROC analysis on all genes in the regulatory axis revealed the potential diagnostic values of hsa_circ_0000417, hsa_circ_0002688, hsa_circ_0001387, hsa_miR-199a-5p, and WDR76 for HCC. However, there were also limitations in our research. This study used public databases, online platforms, and other bioinformatics analyses to determine the value of specific clinical diagnoses and treatments. This study lacked cell and animal experiments to support the findings, which shall be incorporated in future research.

In conclusion, we successfully constructed ceRNA and survival networks by analyzing the differentially expressed circRNAs, miRNAs, and mRNAs. The results reveal an association of the hsa_circ_0000417/hsa_circ_0002688/hsa_circ_0001387-hsa**-**miR**-**199a**-**5p-WDR76 regulatory axis with survival prognosis, tumor-infiltrating immune cells, immune escape, pathway activity, and drug sensitivity in HCC patients. The findings indicate the use of this regulatory axis as new potential biomarkers and targets for the clinical diagnosis and treatment of HCC. The results also provide insights into the mechanisms of HCC development and progression.

## Materials and methods

### Data downloading

The search in the GEO database (http://www.ncbi.nlm.nih.gov/geo/) for the corresponding circRNA, miRNA, and mRNA microarray datasets was conducted by using the keyword “liver cancer”. The criteria for screening and selecting the datasets included the sequence samples containing human liver cancer and its adjacent normal liver tissue. The sample size of the standard and tumor groups was larger than six. GSE155949 (expression profile chip for circRNAs), GSE108724 (expression profile chip for miRNAs), and GSE101728 (expression profile chip for mRNAs) were screened accordingly. The probe matrix and platform files were screened for further analysis. The HCC expression and clinical data were downloaded from TCGA database (https://portal.gdc.Cancer.gov/) for subsequent survival analysis.

### The analysis of differentially expressed genes

Using Perl (version 5.32.1001, https://strawberryperl.com/), the downloaded chip data was sorted out to obtain the corresponding gene expression matrix file. The tumor and the regular groups were assigned according to the probe matrix file. The data were analyzed using R software (version 4.1.2, https://www.r-project.org/) and the ‘Limma’ package, and the adjusted *P* < 0.05 was set as the filtering threshold. Finally, DEcircRNAs, DEmiRNAs, and DEmRNAs of the HCC and normal groups were determined. These DEcircRNAs were used as candidate circRNAs for constructing the ceRNA network. The DEmiRNAs and DEmRNAs were subjected to further analysis.

### The target genes prediction from the database

The targets of DEcircRNAs were predicted using three online databases: CircBanK^44^ (http://www.circbank.cn/), CircRNA Interactome^45^ (https://circinteractome.nia.nih.gov/), and Cancer-Specific CircRNA Database^46^ (http://gb.whu.edu.cn/CSCD/). The candidate miRNAs used for constructing a ceRNA network were obtained by intersecting the targets predicted by the three databases with DEmiRNAs. Target prediction of candidate miRNAs was performed using miRDB^47, 48^ (http://mirdb.org/) and TargetScanHuman^49^ (https://www.targetscan.org/vert_72/) databases. The predicted targets were overlapped with DEmRNAs to obtain candidate mRNAs for constructing the ceRNA network.

### The construction of the ceRNA network

Perl was used to match the relationship between the identified candidate genes to prepare the input files needed to construct the ceRNA network. The relationship between the candidate genes should conform to the ceRNA mechanism as follows: (1) the higher the expression of circRNA, the lower the expression of targeted miRNAs and the higher the expression of downstream mRNAs; (2) the lower the expression of circRNA, the higher the expression of targeted miRNAs and the lower the expression of downstream mRNAs. The ceRNA network was constructed using Cytoscape software^50^ (version 3.8.0, https://cytoscape.org/).

### GO and KEGG enrichment analysis

We used R software and the ‘org.Hs.eg.db’ package to convert the gene symbol of mRNAs in the ceRNA network into the ID required for functional enrichment analysis. And the pathway enrichment analysis was based on KEGG database developed by Kanehisa Laboratories^51–53^. The ‘clusterProfiler, ggplot2, org.Hs.eg.db, enrichplot, circlize, RColorBrewer, dplyr, ggpubr, and complexHeatmap’ packages were applied for GO and KEGG enrichment analysis and data visualization. *P* < 0.05 was used as the screening threshold.

### Survival analysis

The transcriptome and clinical data of hepatocellular carcinoma were retrieved and downloaded from the TCGA database. The data were processed by R and Perl software to obtain the gene expression and survival time file. Survival analysis of mRNAs in the ceRNA network was performed using R software and the ‘survival, survminer’ packages to screen for the mRNAs associated with survival prognosis in HCC patients. The data with a *P* < 0.05 indicated an association between mRNA and the survival prognosis of HCC patients.

### The construction of a survival network

Information on their upstream-related miRNAs and circRNA was obtained using the survival-related mRNAs obtained from the preceding steps. The files of network relationship, node, and other relevant input were prepared accordingly. Cytoscape was used to construct a survival network. The structure, location, and additional information of circRNAs in the survival network were retrieved using circprimer2.0 software^54^.

### Pathway activity analysis

Gene Set Cancer Analysis (GSCALite)^55^ is a cancer genome analysis platform based on a network and multi-database (http://bioinfo.life.hust.edu.cn/web/GSCALite/). Eight highly expressed mRNAs in the survival network pathway were analyzed using the platform. Survival-related genes were entered as required, TCGA expression data were selected, and LIHC was selected for cancer type. The pathway activity analysis module was conducted to analyze and obtain the final results.

### Immune cell infiltration levels and immune checkpoints analysis

Tumor Immune Estimation Resource (TIMER)^56^ is a public network platform (https://cistrome.shinyapps.io/timer/) for analyzing the level of immune cell infiltration in different types of cancer. Eight highly expressed mRNAs in the survival network were selected to analyze the level of immune cell infiltration in hepatocellular carcinoma. These genes were input into the search box, and LIHC was chosen as the cancer type. The immune infiltration-related cells (B cells, CD4+ T cells, CD8+ T cells, neutrophils, macrophages, and dendritic cells) were selected for correlation analysis. Gene Expression Profiling Interactive Analysis (GEPIA)^57^ is a multifunctional online platform (http://gepia.cancer-pku.cn/about.html) for analyzing the differential expression of genes between tumor and normal cells. The platform is also used for survival analysis, similar gene detection, correlation analysis, and dimensionality reduction analysis, etc. related to the chosen cancer type. The correlation analysis module of GEPIA was used in this study to determine the correlation between the expression of the eight mRNAs and three classic tumor immune escape-related immune checkpoints PDCD1, CD274, and CTLA4. LIHC was selected as the cancer type, and Pearson was assigned as the correlation coefficient.

### Drug sensitivity analysis

The GSCALite analysis platform integrates the drug sensitivity and gene expression profile data of cancer cell lines in Genomics of Drug Sensitivity in Cancer (GDSC) and the Cancer Therapeutics Response Portal (CTRP). We performed the drug sensitivity analysis of eight survival-related and highly expressed mRNAs. LIHC was selected as the cancer type, drug sensitivity was selected for the analysis module, GDSC was selected as the cancer drug sensitivity dataset, and Spearman correlation analysis was performed for half maximal inhibitory concentration (IC50). After adjusting the parameters, the results were generated.

### ROC analysis

R software and the ‘pROC’ package were used to perform ROC analysis to clarify the diagnostic value of the genes in the circRNA-miRNA-mRNA regulatory axis for HCC. The expression data from HCC tissues and control groups were analyzed using three microarray data adjusted in the preceding analysis, with AUC ≥ 0.7 considered a diagnostic value for HCC. All statistical analysis was performed using R software or online platforms.

### Confirmation of binding sites and model construction of the regulatory axis

The circprimer2.0 software was used to query the relevant information of the three circRNAs in the regulatory axis and obtain their sequences. The sequence of hsa-miR-199a-5p was downloaded from the miRWalk (version 3, http://mirwalk.umm.uni-heidelberg.de/) and the potential binding sites between the circRNAs and miRNA were found by sequence alignment. Meanwhile, we used the Microsoft PowerPoint software (version 2021, https://www.microsoft.com/zh-cn/microsoft-365/powerpoint) to draw a model diagram of the regulatory axis to show the control relationship between these elements.

### Ethics statement

There was no need for ethical approval since the data used in this study were all from public databases, and we did not conduct any experiments correlated with animals or human specimens.

## Data Availability

The datasets and data used in this work are all available from the GEO (https://www.ncbi.nlm.nih.gov/geo/query/acc.cgi?acc=GSE155949,https://www.ncbi.nlm.nih.gov/geo/query/acc.cgi?acc=GSE108724,https://www.ncbi.nlm.nih.gov/geo/query/acc.cgi?acc=GSE101728) and TCGA databases (https://portal.gdc.Cancer.gov/).
